# Data-Driven UPLC-Orbitrap MS Analysis in Astrochemistry

**DOI:** 10.3390/life9020035

**Published:** 2019-05-02

**Authors:** Alexander Ruf, Pauline Poinot, Claude Geffroy, Louis Le Sergeant d’Hendecourt, Gregoire Danger

**Affiliations:** 1 Laboratoire de Physique des Interactions Ioniques et Moléculaires (PIIM), Université Aix-Marseille, Saint Jérôme—AVE Escadrille Normandie Niemen, 13013 Marseille, France; alexander.ruf@univ-amu.fr (A.R.); ldh@ias.u-psud.fr (L.L.S.d.); 2 Institut de Chimie des Milieux et Matériaux de Poitiers (IC2MP), Université de Poitiers, UMR CNRS 7285, 86073 Poitiers, France; pauline.poinot@univ-poitiers.fr (P.P.); claude.geffroy@univ-poitiers.fr (C.G.)

**Keywords:** astrochemistry, meteorites, high-resolving analytical chemistry, data analysis, origin of life

## Abstract

Meteorites have been found to be rich and highly diverse in organic compounds. Next to previous direct infusion high resolution mass spectrometry experiments (DI-HR-MS), we present here data-driven strategies to evaluate UPLC-Orbitrap MS analyses. This allows a comprehensive mining of structural isomers extending the level of information on the molecular diversity in astrochemical materials. As a proof-of-concept study, Murchison and Allende meteorites were analyzed. Both, global organic fingerprint and specific isomer analyses are discussed. Up to 31 different isomers per molecular composition are present in Murchison suggesting the presence of ≈440,000 different compounds detected therein. By means of this time-resolving high resolution mass spectrometric method, we go one step further toward the characterization of chemical structures within complex extraterrestrial mixtures, enabling a better understanding of organic chemical evolution, from interstellar ices toward small bodies in the Solar System.

## 1. Introduction

Organic chemistry is known to be rich and almost universal in astronomical objects, as observed from a huge molecular diversity in the interstellar medium (ISM) [1], in interstellar/precometary ices of laboratory analogs [2,3,4], in comets [5,6,7] or in meteorites [8,9,10]. When probing different steps in chemical evolution, from simple molecular ices toward complex organic materials, various prebiotically relevant compounds, such as amino acids, sugars or nucleobases are formed in precometay laboratory ices [11,12] or in meteorites [13,14,15]. In addition, organized molecular structures, such as peptides, have been proposed to be present in residues of interstellar ices [16,17,18]. These findings indicate that molecular assembly processes of astrochemical organic matter might have been present within early stages of the Solar System formation. Molecular organization is crucial in early prebiotic phases and might represent a marker to probe the transition between astro-/cosmochemistry and prebiotic chemistry [19,20].

The description of astrochemical organic matter as seeds for life and their interactions within various astrophysical environments may thus appear essential to further study problems regarding the emergence of life, in a given telluric planetary environment [19]. In-depth analysis is essential to understand its evolution, especially for testing the role of extraterrestrial organics in the fields of prebiotic chemistry and astrobiology (e.g., self-organization processes). Besides spectroscopic techniques [21,22,23], mass spectrometry (MS) has been widely used in organic astro- and cosmochemistry over the last several years [19]. MS has also powerfully demonstrated to in situ study the chemical composition of extraterrestrial objects, e.g., for the comet 67P/Churyumov–Gerasimenko [24]. More recently, high resolution mass spectrometric (HR-MS) instruments have had a significant and an ongoing increasing impact in current astrochemical research [25]. For instance, Fourier transform ion cyclotron resonance mass spectrometric (FT-ICR-MS) analyses enabled to resolve mass signals which differ in mass differences by less than the mass of an electron, thanks to resolving power R > 10^6^ and mass accuracy <200 ppb [9]. This type of sophisticated instrumentation allows one to study novel chemistry—as, for example, the one involving organomagnesium compounds in meteorites showing that a rich and complex chemistry has occurred in meteoritic parent bodies [9]. In context of space exploration, another type of high-resolving mass spectrometers, Orbitrap-type instruments, has been optimized in miniaturized form (called “Cosmorbitrap”) [26,27]. Cosmorbitrap instruments could be directly sent on space missions for high resolution mass spectrometric in situ analysis to characterize the chemistry of planetary environments. In nowadays sample return missions (e.g., Hayabusa 2 [28] or OSIRIS-REx [29]), collected extraterrestrial samples are expected to be analyzed as comprehensive as possible. High sensitivity, high selectivity and high resolution should be achieved, but it is also intended to directly elucidate chemical structures. Indeed, most high-resolving mass spectrometric studies on astrochemical organic matter are operated in direct infusion electrospray ionization mass spectrometric mode (DI-HR-MS) [2,3,4,8,9]. By means of this technique, powerful insights on separate molecular formulas (isobars and isotopologues) are given, but no information on structural isomers can be obtained therewith [30,31].

By coupling chromatography with HR-MS instruments (e.g., liquid chromatography, LC-MS), the level of information on astrochemical complex mixtures would be increased [32]. Whereas mass spectrometry represents a physical analytical technique (determining the weight of a molecule), chromatography directly provides insights into chemical properties. The temporal dimension (retention time) gives information on the polarity of a molecule, by means of understanding the chemical interactions between analyte molecules and the chromatography’s stationary phase. Thus, molecular isomers (compounds with the same exact molecular masses but different structures) can be distinguished [30,33]. The discrimination of isomers is not possible with DI-HR-MS only.

LC-HR-MS analyses generate information-rich and high-dimensional datasets that remain challenging to handle and to evaluate in a global, nontargeted manner [34]. This makes it difficult to describe a complex chemical fingerprint in a comprehensive manner. In complex astrochemical mixtures, each chromatographic data point represents a high resolution mass spectrum out of ten thousands of detected *m*/*z* signals. Thus, a proper data analytical methodology is critical but also crucial to generate meaningful results [19]. By the help of sophisticated data mining, these types of large, multivariate big data sets can be practically accessed. Furthermore, proper data visualization tools are required for interpretation and first-level comparison of different samples analyzed by high-resolution mass spectrometry [35,36].

Here, we will go one step further toward the analysis of chemical structures in complex astrochemical mixtures. Data-driven ultra high performance liquid chromatography (UPLC)-Orbitrap MS analysis of Murchison (CM2) and Allende (CV3) meteorite will be discussed. By that, we add a new analytical dimension (chromatography) to enable the mass spectrometric detection of isomers, next to the resolution of isobars by HR-MS. In this proof-of-concept study, we will (i) give the motivation of using LC-HR-MS in astro-/cosmochemistry, (ii) discuss different chemical families in Murchison and (iii) give a first-order comparison between Murchison and Allende soluble organic matter regarding their chemical isomers. Our aim is to develop a conceptual overview on the multi-level information provided by comprehensive UPLC-HR-MS analysis.

## 2. Results

### 2.1. Complexity of Astrochemical Organic Matter—The Need for Additional Analytical Dimensions

**Figure 1:**
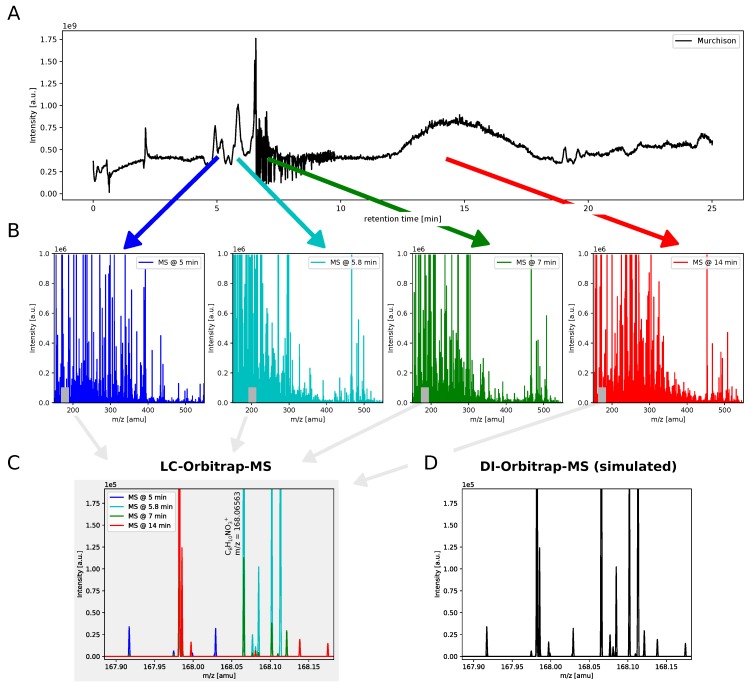
Complexity of astrochemical organic matter. (**A**) Positive ESI mode total ion current (TIC) 1D chromatogram of Murchison soluble organic matter; (**B**) different retention times result in different mass spectrometric fingerprints between 150 and 550 amu (atomic mass units); (**C**) the nominal mass *m*/*z* = 168 bears 17 different *m*/*z* signals, whose intensities vary for different retention times; (**D**) a simulated DI-Orbitrap mass spectrum of *m*/*z* = 168 amu doesn’t give information on structural isomers. In the simulated DI mass spectrum, all mass spectra along the chromatographic run were averaged.

Extraterrestrial organic matter is highly diverse, likely resulting in millions of molecules [8,19,37]. 14,000 various molecular formulas were described, including up to 150 different signals of molecular compositions per nominal mass [8]. Nevertheless, these type of DI-HR-MS experiments only determine a molecular composition but do not give any insights on the presence of isomers or chemical structures in general (without MS/MS). Complementary to DI-HR-MS analyses [8,9], we analyze here Murchison soluble organic matter by UPLC-Orbitrap MS. The total ion current (TIC) 1D chromatogram is rich in signals which reflects the overall molecular diversity present in Murchison (Figure 1A).

By adding the LC dimension to HR-MS, intensities of *m*/*z* signals vary for different retention times in a broad mass range between 150 and 550 amu (atomic mass units, Figure 1B). This gives first insights into (i) the presence of isomers (intensity variation for the same exact mass in different time-resolved mass spectra, Figure 1C) and (ii) a general trend onto the relative chemical polarity scale of molecules (involving the identification of homologous series). Signals with small retention times bear higher polarity compared to the ones eluting at the end of a chromatographic run.

Thanks to chromatographic separation, the presence of isomers was elucidated in detail. As an example, for *m*/*z* = 168 amu, 17 different *m*/*z* signals could be detected (Figure 1C). The intensities of these mass peaks vary for different retention times. Thus, each *m*/*z* value corresponding to a molecular formula consists of various isomers. For example, *m*/*z* = 168.06549 corresponds to C_8_H_10_NO_3_^+^. Thirteen different isomers for this molecular formula have been detected in Murchison soluble organic matter. Because of their difference in chemical polarity, different isomers could be separated on a chromatographic column, while HR-MS only provides the resolution of isobars.

In comparison, direct infusion mass spectrometric (DI-HR-MS) experiments don’t give any information on isomers (Figure 1D), as shown in the simulated direct infusion (DI) mass spectrum. The simulated DI mass spectrum was constructed by averaging all mass spectra along the chromatographic run. Thus, information on the retention time is vanished and only the differentiation of *m*/*z* signals (assignable to molecular compositions) remains. This directly demonstrates the importance of coupling LC to HR-MS.

In summary, DI-HR-MS gives the first level of information on the molecular diversity but only the coupling of MS with LC can describe isomers distinctly. This considerably increases our view onto the molecular diversity present in complex extraterrestrial samples.

### 2.2. Classifying Chemical Families—Bringing Order into the Chemical Diversity

**Figure 2:**
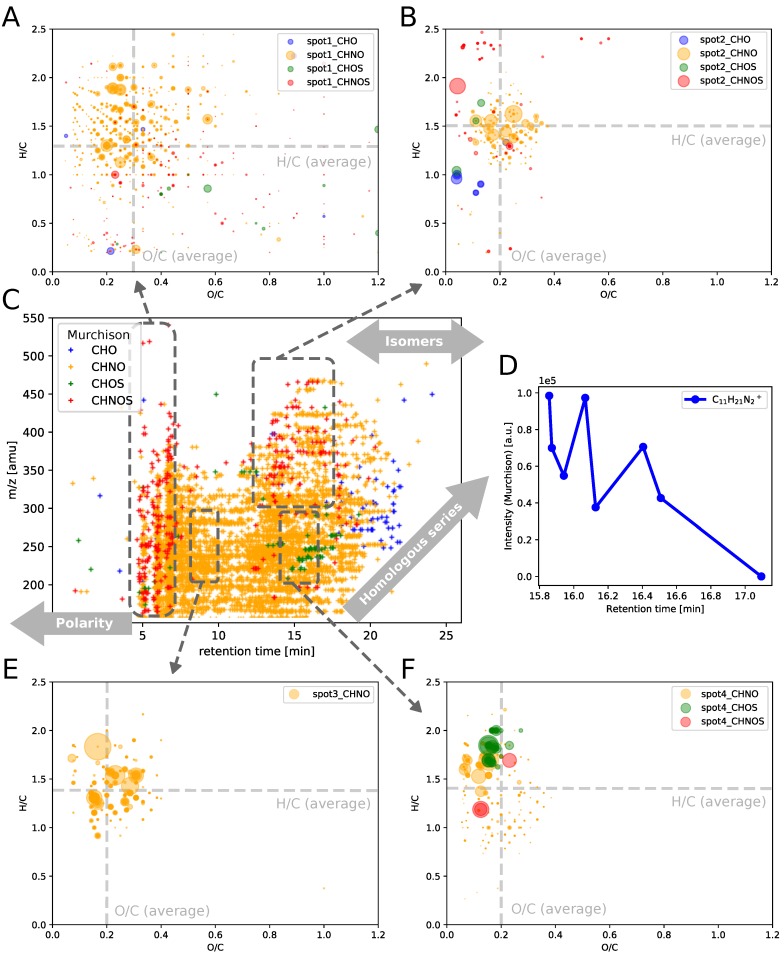
Classifying chemical families. (**A**) van Krevelen diagrams of spot 1 (RT = 4–7 min, *m*/*z* = 150–700 amu); (**B**) van Krevelen diagrams of spot 2 (RT = 12.5–17.5 min, *m*/*z* = 300–500 amu); (**C**) 2D chromatogram (*m*/*z* over retention time) of Murchison soluble organic matter depicts its chemical diversity in these two dimensions but also shows the grouping of distinct chemical subspaces CHO, CHNO, CHOS and CHNOS; (**D**) the resolved detection of eight C_11_H_21_N_2_^+^ isomers in Murchison gives first insights on the different chemical polarity of these compounds. (**E**) van Krevelen diagrams of spot 3 (RT = 8–10 min, *m*/*z* = 200–300 amu); (**F**) van Krevelen diagrams of spot 4 (RT = 14–17 min, *m*/*z* = 200–300 amu). Bubble sizes in van Krevelen diagrams are scaled with mass spectrometric intensites.

The 2D chromatogram of Murchison soluble organic matter (*m*/*z* over retention time, Figure 2C), is rich in signals between *m*/*z* = 150–350 amu and from retention time = 5–20 min. Dealing with such a high degree of molecular diversity, it is important to systematically group chemical spaces. Here, chemical families were classified into CHO, CHNO, CHOS and CHNOS within LC-HR-MS data of Murchison. CHO, CHNO, CHOS and CHNOS compounds show different characteristics, both in *m*/*z* and in retention time (Figure 2C). This gives hints on chemical characteristics for each chemical class. In general, information on chemical polarity, structural isomers and homologous series can be obtained from this raw data representation. CHNO compounds represent the largest chemical subspace, distributed along the whole mass and retention time range which is in agreement with previous studies [3,8,10]. The significance of nitrogen chemistry in meteorites has been previously reported [38,39,40,41,42,43,44,45]. Otherwise, CHNOS compounds were found to group at fairly small retention times. This indicates a higher polarity of CHNOS species, compared to CHO, CHNO or CHOS classes. The contrary effect has been observed for CHO and CHOS compounds which elute toward the end of a chromatographic run suggesting more apolar properties.

Specific *m*/*z* vs. retention time windows were elucidated in more detail. Four spots were sampled and result in different van Krevelen diagrams (Figure 2A,B,E,F). Spot 1 represents molecules of highest polarity in Murchison (Figure 2A). They range from *m*/*z* 150–550 amu and mostly consist of CHNO and CHNOS species. Chemically, they show a high diversity in the degree of unsaturation (H/C ratio) indicating the presence of both aliphatic and unsaturated structures. In addition, the degree of oxygenation (O/C ratio) varies from 0.1 until 1.2. In comparison to spot 1, spot 2 represented more apolar compounds at high molecular masses (300–500 amu, Figure 2C). Chemical polarity can scale with the degree of oxygenation. This is reflected by a less widespread O/C ratio of spot 2 compounds when compared to those in spot 1 (O/C (average, spot 1) = 0.3, O/C (average, spot 2) = 0.2, Figure 2A,B). In addition, the H/C ratio is also narrower for higher apolar compounds (spot 2) in contrast to spot 1 compounds (H/C (average, spot 1) = 1.3, H/C (average, spot 2) = 1.5, Figure 2A,B). Spot 3 and spot 4 represent the same *m*/*z* range (200–300 amu) but differ in their retention time windows (Figure 2C). Spot 4 elutes later in the chromatographic run (higher retention time), thus indicating the presence of more apolar compounds. van Krevelen diagrams represent similar chemical characteristics for both molecular subsets (Figure 2E,F). These two chemical subspaces consist of many structural isomers (same *m*/*z* value but different retention time) which cannot be discriminated in terms of van Krevelen-type representations (atomic ratio plots).

In-depth analyses on the number of isomers show that Murchison soluble organic matter bears a high number of isomers per molecular composition. As an example, eight different isomers of C_11_H_21_N_2_^+^ molecular compositions could be chromatographically resolved and separately detected. Different C_11_H_21_N_2_^+^ isomers showed variations in their detected mass spectrometric (+)-ESI intensities (Figure 2E). This intensity distribution might indicate differences in isomer stabilities (assuming similar ionization efficiency of the respective isomers).

These results underline the importance of studying isomers to get further insights into chemical structures in complex astrochemical mixtures. In the next section, we further study the isomeric diversity of Murchison and Allende in a more comprehensive manner.

### 2.3. Comparison of Murchison with Allende—A Difference in Isomeric Diversity

To illustrate the power of comprehensive UPLC-Orbitrap MS analyses in complex extraterrestrial mixtures, we compared Murchison with Allende meteorite in a coarse, first-order manner. Murchison represents a primitive CM2 carbon-rich carbonaceous chondrite [46]. It has become a reference material for extraterrestrial organic matter [8,10,13,47,48]. This CM2-type meteorite has been reported to have experienced weak secondary, aqueous alteration processes [49,50]. Allende (CV3 chondrite) is assigned as a petrologic type 3 meteorite and has undergone thermal metamorphism, indicating a modification/alteration of its organic matter [51]. Compared to Murchison, Allende has lower organic contents [52]. Anyhow, a rigorous direct comparison between Murchison and Allende in terms of chemical evolution is difficult. Both objects are supposed to have different parent bodies and might be different in their primary organics.

**Figure 3:**
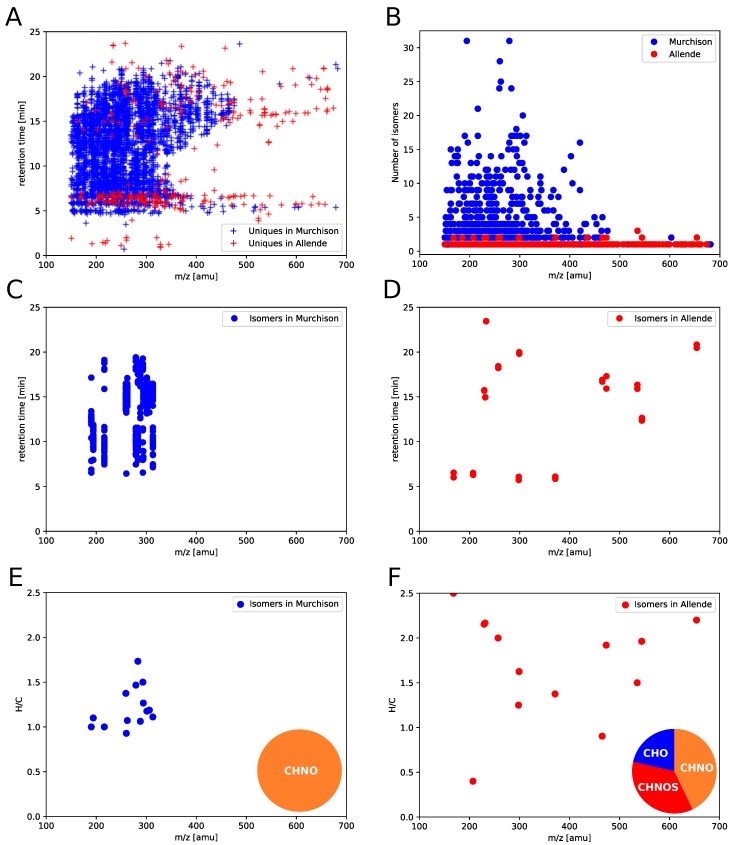
Comparison of Murchison with Allende. (**A**) 2D chromatogram highlighting the compound distributions of Murchison and Allende; (**B**) number of isomers as a function of *m*/*z*; (**C**,**D**) 2D chromatogram of the top 15 molecular compositions sorted by the number of isomers in Murchison (**C**) and Allende (**D**); (**E**,**F**) mass-edited van Krevelen diagrams of the top 15 molecular compositions sorted by the number of isomers in Murchison (**E**) and Allende (**F**). In all plots, only unique features present in Murchison or Allende, respectively.

The molecular diversity of Murchison soluble organic matter rules the overall chemical space of the two samples (blue crosses, Figure 3A). Allende soluble organic matter seems to be more diverse in *m*/*z* range (red crosses, Figure 3A). Whereas Murchison organic matter shows a continuum in chemical polarity, Allende is characterized by more distinct properties. Allende bears either fairly polar molecules (retention time 5–7 min) or apolar compounds, widespread over a large mass range (retention time 14–24 min, *m*/*z* 150–600 amu).

The unique power of UPLC-Orbitrap MS analyses is the combination of accurate mass measurement with resolving structural isomers. By monitoring isomer counts for both Murchison and Allende meteoritic soluble organic matter, a significant difference in isomeric diversity has been revealed (Figure 3B). Unique features in Murchison are present with a maximum of 31 different isomers. The number of isomer counts decreases exponentially which might indicate a statistical process in the diversification of molecular structures (Supplementary Materials, Figure S3). Allende is much less diverse in isomers than Murchison. Only up to three different isomers were found herein. Both isomeric distributions differ in their mass ranges. Most Murchison isomer candidates were found at small *m*/*z* values (200–300 amu). In contrast, Allende isomers were present over a larger mass scale (350–650 amu). Based on this finding, ≈440,000 different organic compounds could be present in Murchison. The estimated number of 440,000 different compounds is based on the following extrapolation: 14,197 molecular compositions [8] × 31 different isomers.

The top 15 molecular compositions with the highest diversity in isomers have both large and small differences in respective retention times (Figure 3C,D). Herein, the top 15 molecular compositions sorted by the number of isomers are depicted. Both isomeric sets of Murchison and Allende reflect different chemical characteristics (Figure 3E,F). Murchison isomers show more similar properties than those of Allende. In Murchison, the extracted top 15 compositions, representing the most diverse isomers, are exclusively CHNO compounds in a fairly narrow H/C and *m*/*z* range (H/C = 1–1.7, *m*/*z* = 180–300 amu). On the contrary, Allende isomers are widespread from H/C = 0.5–2.5 and *m*/*z* = 200–700 amu. In addition, Allende isomers consist of CHO, CHNO and CHNOS molecules.

Murchison and Allende are significantly distinct in their sets of chemical isomers. Murchison bears a significant higher number of isomers, while their chemical characteristics seem to be less diverse than those of Allende. This finding might give hints on the different secondary processing (aqeuous alteration/thermal metamorphism) in Murchison and Allende and/or in their parent bodies. These findings are consistent with previous studies, indicating an inverse relation between molecular diversity and degree of meteoritic alteration [8,9,44].

## 3. Materials and Methods

### 3.1. Extraction of Meteorites

All chemicals were purchased from Sigma-Aldrich, Steinheim, Germany. Purified water was generated by a Purelab UHQ II water purification System (ELGA Lab Water, Veolia, Paris, France). Both meteorites were provided by Musée Cantonal de Géologie (Lausanne, Switzerland). Glassware used in sample processing were pyrolyzed at 500 °C in air overnight prior to use. For both Murchison and Allende, a sample (1.5 g) was sonicated with water (2.5 mL) at 50 °C for 20 h. The resulting solution was recovered by centrifugation (4500 rotations per minute (rpm), 10 min) and discarded. The sample (approximately 500 mg) was then powdered in an agate mortar and extracted with water (2 mL) at 100 °C for 20 h in an evacuated and N${}_{2}$ purged vial. After centrifugation, the supernatant was removed, dried down with a speedvac and resuspended in water (2 mL). One millilitre aliquot of the resuspended sample was desalted through a cation-exchange resin (Dowex 50W-X8 resin, 50–100 mesh, hydrogen form) and the eluates of ammonium hydroxide solution were collected and dried under vacuum. It was stored at −20 °C until use. It was dissolved in 100 µL of purified water and diluted in methanol (1/3) for LC-MS analysis.

### 3.2. UPLC-Orbitrap MS Analysis

Analyses were performed with two quaternary Accela LC pumps (pump 1: Accela 600 pump; pump 2: Accela 1250 pump) working together and interfaced with a Q-Exactive (Hybrid quadrupole-OrbitrapTM) mass spectrometer equipped of an HESI source (Thermo Fisher Scientific, Waltham, MA, USA). Before injection, samples were stored at 4 °C using a Stack cooler CW (CTC Analytics AG, Zwingen, Switzerland). MS functions and LC solvent gradients were controlled by the Xcalibur data system (Thermo Fisher Scientific). Acetonitrile with 0.1% formic acid was used as buffer A and water with 0.1% formic acid as buffer B. Instrument calibration in positive mode was done every day via the direct infusion of positive ions calibration solutions. Compounds elution was performed via a Dual-LC configuration composed of two distinct units that are each composed of two stationary phases coupled in series. Unit 1 corresponds to the dual-trap unit. It combines a semi-polar Hypersil Gold aQ (20 mm × 2.1 mm, 12 µm, 175 Å; Thermo Fisher Scientific) and a porous graphitic carbon Hypercarb (20 mm × 2.1 mm, 7 µm, 250 Å; Thermo Fisher Scientific). They are connected to a second analytical unit which combines a Pentafluorophenyl reverse-phase Kinetex F5 (100 mm × 2.1 mm, 1.7 µm, 100 Å, Phenomenex) and a C18 reverse-phase Hypersil Gold Q (50 mm × 1 mm, 1.9 µm, 175 Å, Thermo Fisher Scientific)). One thousand microliters of the sample are first transferred to the dual trap unit with water—0.1% formic acid for 2 min at 100 µL·min${}^{-1}$. Once the loading completed, the second elution phase (5% buffer A and 95% buffer B) passes through the whole system for 4 min at 100 µL·min${}^{-1}$. Trapped analytes are then refocused and eluted into the serially coupled columns Kinetex F5—Gold Q in a backflush mode to narrow the peaks. Molecules are separated in the series of analytical columns through two successive gradient steps; the first one reaching 30% buffer A and 70% buffer B in 4 min and the second one reaching 100% buffer A in 12 min. The columns are then rinsed with 100% buffer A during 3 min and reconditioned for 4 min with 5% buffer A and 95% buffer B.

Mass detection was performed in positive ion mode with a full scan mode with a resolution of 140,000. Electrospray voltage was set at 4.0 kV. Capillary and heater temperatures were 275 °C and 300 °C. The relative flow rate of the sweep gas (nitrogen) which aids in solvent declustering and adduct reduction was set to 10 (flow in arbitrary units as defined by Thermo Fisher Scientific). To help nebulise sample solution into a fine mist in the ESI nozzle, the relative flow rate of the sheath gas (nitrogen) was set to 35 (flow in arbitrary units as defined by Thermo Fisher Scientific). Finally, the relative flow rate of the auxiliary gas (nitrogen) which assists the sheath gas in dispersing and/or evaporating sample solution was set at 20 (flow in arbitrary units as defined by Thermo Fisher Scientific). Instrument calibration in positive and negative mode was done every 3 days via the direct infusion of positive and negative ion calibration solutions. Peaks integration and MS spectra acquisition were performed with Thermo XcaliburTM Qualitative Browser (Thermo Xcalibur 2.2 SP1.48, Thermo Fisher Scientific Inc.).

We have used solvent blanks which signatures were subtracted from endogenous analyte signals from each sample (Murchison and Allende). The method used here was optimized to study the molecular diversity of astrochemical samples [53].

### 3.3. Data Analysis

**Figure 4:**
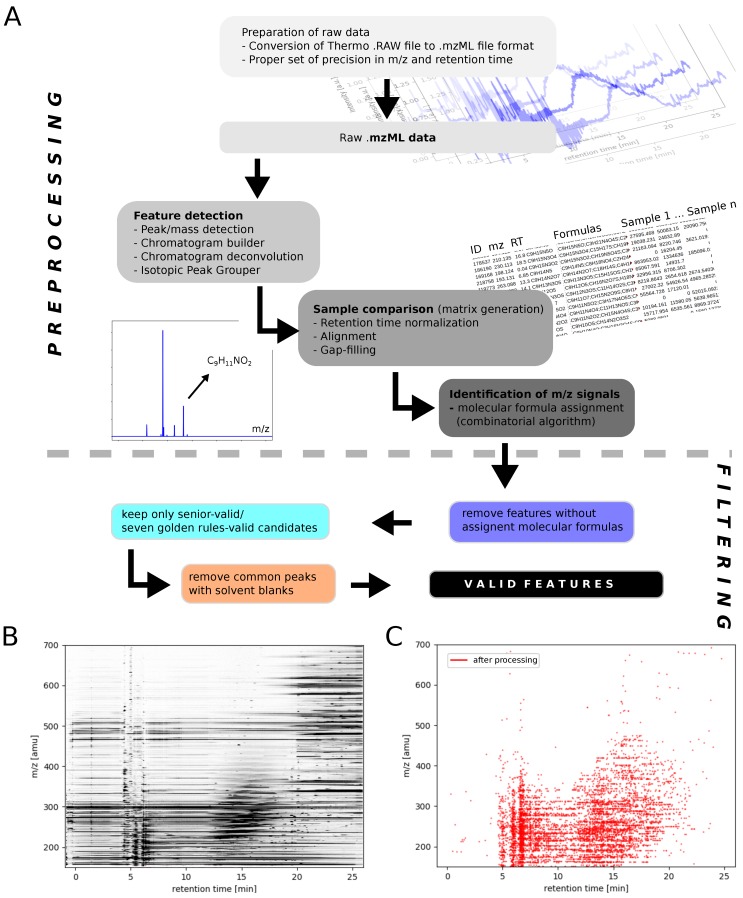
Data processing workflow—a protocol to merge highdimensional LC-HR-MS data. (**A**) the filtering and optimized process of this data set reduced the data set from ≈400,000 features to ≈7000 features; (**B**,**C**) *m*/*z* over retention time plot shows the difference in *m*/*z* signals before and after processing.

The whole LC-HR-MS data analysis workflow (Figure 4) was performed using MZmine 2.37 [54]. All computations were done on a stand-alone computer. Further processing (calculations and filtering) were done in Python and in LibreOffice Calc. The major part of this work is expressed by handling the complexity of LC-HR-MS big data sets. It is a challenge to mine these highdimensional data resulting from ≈40,000 *m*/*z* signals per mass spectrum, multiplied by ≈2600 chromatographic data points, multiplied by the number of samples (here 3, Blank, Murchison and Allende). Thus, ≈3 × 10${}^{8}$*m*/*z* signals need be handled. The treatment is problematic, both in computational costs and in terms of error propagation. Thus, a strict validation procedure to test for errors along the whole data analytical workflow is absolutely necessary. The preprocessing procedure (raw file toward unfiltered matrix) was tested to three types of errors; (i) errors in *m*/*z* during matrix generation, (ii) errors in retention time (RT) during matrix generation and (iii) error in *m*/*z* during molecular formula assignment. Errors in *m*/*z* (no i and iii) are mainly within ± 2.5 ppm (Supporting Materials, Figures S1 and S2). Thus, a *m*/*z* tolerance of 5 ppm within the whole workflow is justified. Within formula assignment, 54.4% of all formulas have an error of ±0.2 ppm, 90.3% of formula assignments within ±0.5 ppm and 99.6% of all formulas within an error window ±1 ppm.

The data analytical workflow consists of two main parts, (i) a preprocessing step and (ii) a filtering step (Figure 4). First, Thermo RAW files were converted via ProteoWizard msconvert into a mzML format [55,56]. Then, mzML data were processed in MZmine 2.37 [54]. Within MZmine, the workflow can be mainly divided into three steps, (i) feature detection, (ii) sample comparison and (iii) identification of *m*/*z* signals. The finalized preprocessing procedure ended in a matrix including 429,040 features (Figure 4B). This first part of analysis was tested to three types of errors; (i) errors in *m*/*z* during matrix generation, (ii) errors in retention time (RT) during matrix generation and (iii) error in *m*/*z* during molecular formula assignment. Details are given in the Supporting Materials section. The second main part of the data processing workflow, filtering invalid features, consisted of four steps: (i) not assigned *m*/*z* features (e.g., noise peaks); (ii) testing for the validation of a molecular formula according Senior’s rules [57] and with respect to the Seven Golden Rules [58]; (iii) remaining features were removed which were in common with solvent blanks. Finally, 6760 valid features containing endogenous signals from Murchison and Allende remained for further analysis (Figure 4C).

## 4. Conclusions

We present in-depth data-analytical strategies to comprehensively evaluate UPLC-Orbitrap MS analysis. As a proof-of-concept study, we studied Murchison and Allende meteorites. These findings indicate the power of this type of analysis but also state the challenge in terms of big data processing.

In summary, (i) LC-HR-MS extends the level of information of astrochemical molecular diversity, (ii) a relative polarity scale of compounds out of complex mixtures can be determined, (iii) structural isomers can be resolved by chromatography and separately detected; (iv) and the combination of UPLC and HR-MS merges both accurate isomer and isobar detection.

By means of this time-resolving high resolution mass spectrometric method, we go one step further toward the characterization of chemical structures within complex extraterrestrial mixtures, enabling a better understanding of organic chemical evolution.

## Supplementary Materials

The following are available online at https://www.mdpi.com/2075-1729/9/2/35/s1, Table S1: MZmine workflow parameters, Figure S1: Error in m/z during matrix generation, Figure S2: Error in retention time (RT) during matrix generation, Figure S3: Frequency distribution of the number of isomers (per molecular formula) present in Murchison and Allende.
