# Supplementary material for: Data-Driven UPLC-Orbitrap MS Analysis in Astrochemistry
Source: Life (Basel). 2019 May 2;9(2):35. doi: 10.3390/life9020035 (PMC6617268; doi:10.3390/life9020035)
Supplement: Supplementary file 1 [file life-09-00035-s001.pdf]

life

Article

Data-Driven UPLC-Orbitrap MS Analysis in Astrochemistry

Alexander Ruf [1,*], Pauline Poinot [2], Claude Geffroy [2], Louis Le Sergeant d'Hendecourt [1] and Gregoire Danger [1,*]

[1] Laboratoire de Physique des Interactions Ioniques et Moléculaires (PIIM), Université Aix-Marseille, Saint Jérôme—AVE Escadrille Normandie Niemen, 13013 Marseille, France; alexander.ruf@univ-amu.fr (A.R.); ldh@ias.u-psud.fr (L.L.S.H.); gregoire.danger@univ-amu.fr (G.D.)

[2] Institut de Chimie des Milieux et Matériaux de Poitiers (IC2MP), Université de Poitiers, UMR CNRS 7285, 86073 Poitiers, France; pauline.poinot@univ-poitiers.fr (P.P.); claude.geffroy@univ-poitiers.fr (C.G.)

* Correspondence: alexander.ruf@univ-amu.fr (A.R.); gregoire.danger@univ-amu.fr (G.D.); Tel.: +33-491288285 (G.D.)

Received: date; Accepted: date; Published: date

SUPPLEMENTARY MATERIALS

Table S1. MZmine workflow parameters. Reference for msconvert and MZmine are given in the main text.

Function	Parameter	Value
raw -> mzml (via msconvert)		
raw data import in MZmine 2.37		
raw data preparation	precision in m/z	5 decimal places
	precision in retention time	2 decimal places
	precision in intensity	2 decimal places
peak detection → mass detection	local maxima mass detector	noise level = 1e0
chromatogram builder	min time span (min)	0.03
	min height	1e3
	m/z tolerance	0.001 / 5 ppm
deconvolution of chromatogram	local minimum search	
	chromatographic threshold	1%
	search minimum in RT range (min)	0.03
	minimum relative height	1%
	minimum absolute height	1e3
	min ratio of peak top/edge	2
	peak duration range (min)	0-10
	m/z center calculation	median
isotopic peaks grouper	mz tol	0.001 / 5 ppm
	ret time tol	0.1 min
	max charge	1
	repr. isotope	most intense
retention time normalizer	mz tol	0.001 / 5 ppm
	ret time tol	0.1 min
	min stand int	1e3
ransac aligner	mz tol	0.001 / 5 ppm
	rt tol	0.15 min
	rt tol after corr	0.15 min
	ransac iterations	1000
	minimum number of points	20%
	threshold value	0.03 min
	require same charge state	
gap filling - same rt and mz range gap filler	mz tol	0.001 / 5 ppm
id - formula prediction	charge	+1
	ionization type	[M+H]+
	mz tol	0.001 / 5 ppm
	max best formulas per peak	10
	min	S0C0O0H0N0
	max	S3C100O100H100N5

© 2019 by the authors. Licensee MDPI, Basel, Switzerland. This article is an open access article distributed under the terms and conditions of the Creative Commons Attribution (CC BY) license (http://creativecommons.org/licenses/by/4.0/).

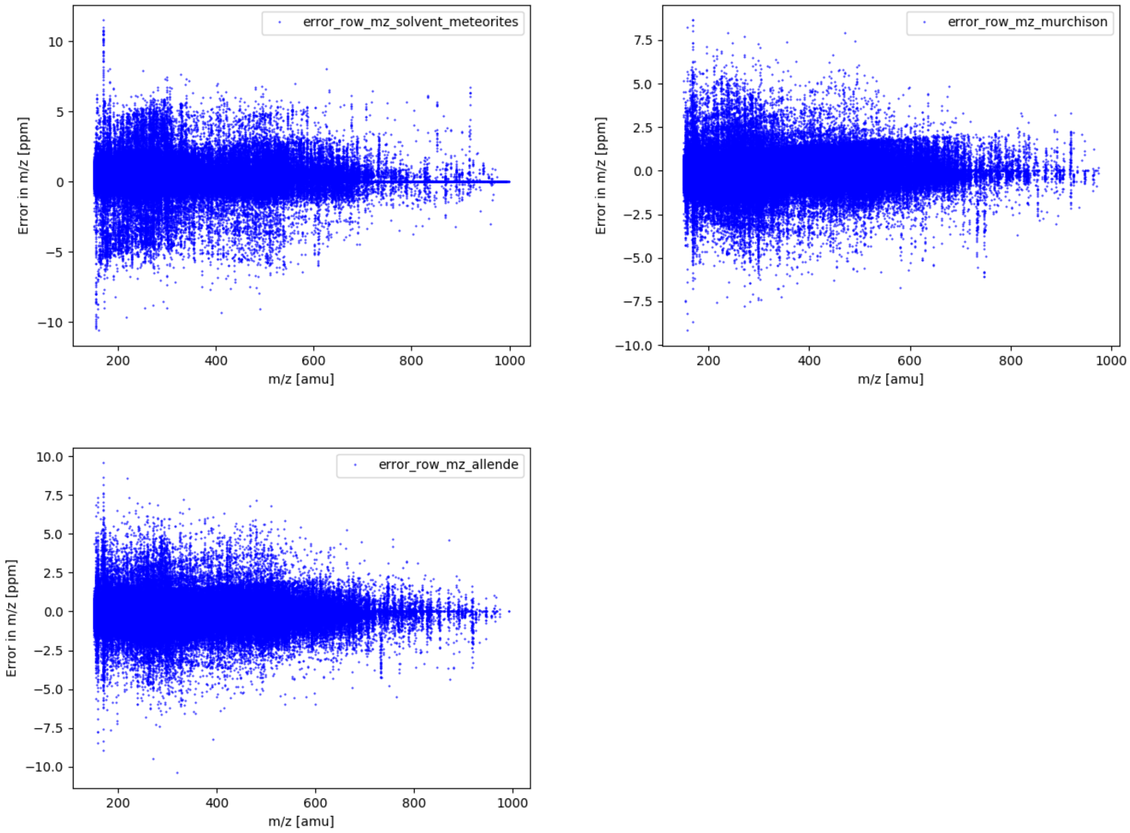

Figure S1. Error in m/z during matrix generation. Errors are majorly referred to the alignment process in MZmine. The error was calculated by (m/z merged matrix - m/z in sample) / m/z merged matrix * 1e6) for each row/feature.

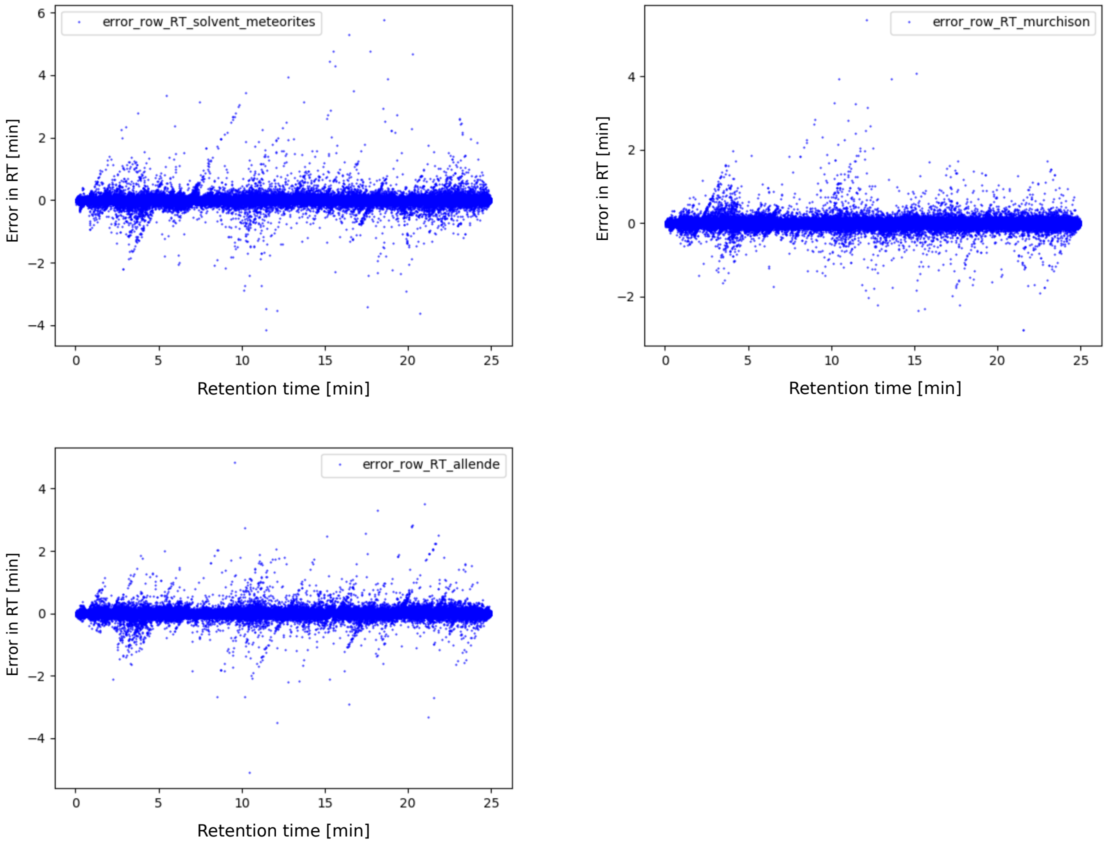

Figure S2. Error in retention time (RT) during matrix generation. Errors are majorly referred to the alignment process in MZmine. The error was calculated by RT merged matrix - RT in sample for each row/feature, with RT as retention time.

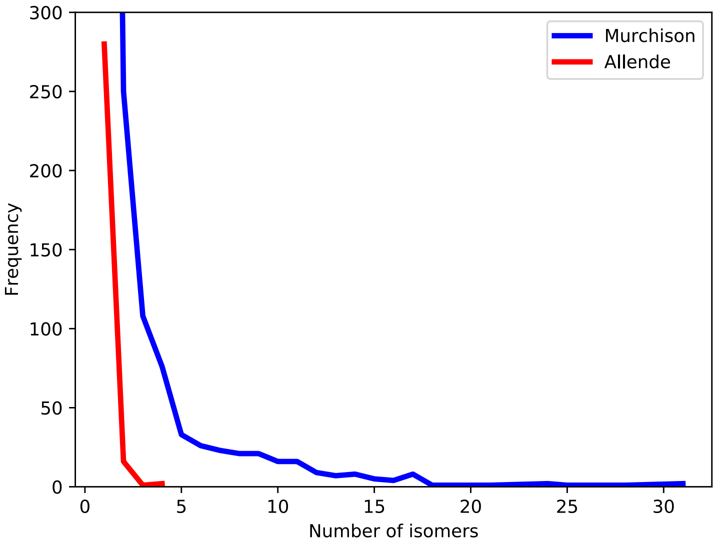

Figure S3. Frequency distribution of the number of isomers (per molecular formula) present in Murchison and Allende.
